# Camera Trap Design Determines Taxa Detected at Carrion Sites

**DOI:** 10.1002/ece3.73735

**Published:** 2026-06-05

**Authors:** Annesha Lahiri, Kathleen A. Carey, Marcus A. Lashley, Brandon T. Barton, Michael V. Cove, Carolina Baruzzi

**Affiliations:** ^1^ Department of Wildlife Ecology and Conservation, Institute of Food and Agricultural Sciences University of Florida Gainesville Florida USA; ^2^ Department of Wildlife Ecology and Conservation, Institute of Food and Agricultural Sciences, North Florida Research and Education Center University of Florida Quincy Florida USA; ^3^ Bioenvironmental Monitoring and Assessment Program, Trent University Trent University Peterborough Ontario Canada; ^4^ North Carolina Museum of Natural Sciences Raleigh North Carolina USA; ^5^ Department of Wildlife, Fisheries, and Aquaculture Mississippi State University Starkville Mississippi USA

**Keywords:** food web monitoring, scavenger, trophic interactions, vulture

## Abstract

Camera traps are a widely used tool in ecology to study vertebrate species and are increasingly used to study invertebrate species. However, variations in camera trap design may influence species detection and, therefore, impact the accuracy of monitoring results. Understanding potential detection biases relative to different camera trap designs is essential to account for such biases, especially in cases where both vertebrates and invertebrates influence food webs. Carrion decomposition can be driven by both animal groups. While previous research has focused on improving the camera trap design for studying either vertebrates or invertebrates in other systems, it is largely unknown which design is most effective at capturing the full carrion food web across trophic levels. We conducted an experiment at Panther Swamp National Wildlife Refuge, MS, USA, comparing two different camera trap designs (i.e., horizontal and vertical) across 17 carrion deployments to study whether the two camera designs produced different estimates of taxonomic richness, community composition, and taxonomic associations. Although the two camera designs produced similar taxonomic richness estimates, the community composition was significantly different. Notably, the vertical design was more likely to detect necrophagous invertebrates (i.e., blow flies [*Calliphoridae*] and carrion beetles [*Silphidae*]), while the horizontal design was more likely to detect white‐tailed deer [ *Odocoileus virginianus* ], suggesting that a broader camera range increases detections of non‐scavenger species. Our study demonstrates a detection bias based on camera trap design, therefore, pairing horizontal and vertical camera traps may increase the breadth of taxonomic groups captured interacting with the carrion food web.

## Graphical abstract

In this study, we demonstrated that camera trap orientation (i.e., horizontal and vertical) at carrion sites differed in their animal community composition and taxa associations. The vertical camera trap orientation was more likely to detect necrophagous invertebrates, while the horizontal orientation was more likely to detect white‐tailed deer. These findings underscore the importance of camera orientation when designing studies aimed at capturing trophic interactions.

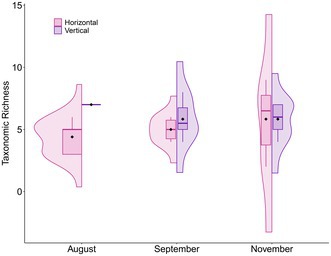

## 1 Introduction

Camera trapping is a noninvasive, affordable method for studying various aspects of wildlife ecology, including species occupancy and abundance (Wevers et al. 2021; Twining et al. 2024), activity patterns (Lashley, Cove, et al. 2018; Lashley, Jordan, et al. 2018; Dykstra et al. 2023; Iannino et al. 2025), and species interactions (Selwyn et al. 2020; Baruzzi et al. 2022). More recently, camera traps have been applied to invertebrates, particularly when identifying species at higher taxonomic levels (e.g., order) is sufficient to meet research objectives (Preti et al. 2021; Seimandi‐Corda et al. 2024). As camera traps become increasingly popular for observing invertebrates, determining optimal designs for these smaller taxa is essential.

Camera trap design can influence species detectability (Smith and Coulson 2012; Taylor et al. 2013; Nichols et al. 2017; Moore, Valentine, et al. 2020). For instance, horizontal camera traps are extensively used due to their wide field of view, which ensures the detectability of various vertebrates (Meek et al. 2016; Nichols et al. 2017). While less common, vertical and slanted camera designs are typically used to monitor a defined focal area. For example, these designs have been used to study nest behavior (Dedieu et al. 2023), artificial nest predation (Palencia and Barroso 2024), and wildlife feeding preferences (Boggess et al. 2022; McDonald et al. 2023). Vertical and slanted designs have also been used to monitor invertebrate taxa (Zeitler et al. 2023; Gao et al. 2024). Previous studies comparing camera trap design suggest that species size influences detectability and thus should inform camera trap design selection (Taylor et al. 2013; Nichols et al. 2017). Smaller taxa, such as small mammals, reptiles, and insects, may be easier to detect with a vertical design because of methodological constraints of horizontal designs, which can be more sensitive to placement error (e.g., height and alignment). This can create gaps in the detection zone and increase the likelihood of missed detections for small species. They also require larger cleared areas, increasing the risk of vegetation obstruction. Vertical cameras are generally less prone to misalignment and provide a more direct, concentrated view of a target area (Smith and Coulson 2012). As such, choosing a camera trap design capable of detecting a range of species sizes may allow us to observe a more complete set of species interactions, therefore improving our understanding of complex food webs.

Dead animal matter is an important resource in terrestrial and aquatic ecosystems (Barton and Bump 2019). Carrion is exploited by a variety of vertebrates and invertebrate species (DeVault et al. 2003; Barton et al. 2013), and scavenger activity at carrion sites can influence both decomposition rates and community dynamics (Wilson and Wolkovich 2011). This highlights the importance of understanding interactions across multiple scales within carrion food webs (Hill et al. 2018; Baruzzi et al. 2018), as these community dynamics are a key knowledge gap in carrion ecology (Barton et al. 2013, 55). For example, smaller consumers may be more likely to be detected with a vertical or modified design compared to the traditional horizontal one, while the latter may be more likely to detect their predators (e.g., blow flies [*Calliphoridae*] and nine‐banded armadillos [*Dasypus novencinctus*]; Lashley, Cove, et al. 2018; Lashley, Jordan, et al. 2018). Variations in camera trap design may influence which taxonomic groups are detected in the carrion community. Identifying these potential variations in detections based on the camera design may allow researchers to account for such biases, thus establishing a more accurate assessment of processes such as resource partitioning and decomposition (Newsome et al. 2021; von Hoermann et al. 2021; Hashizume et al. 2023).

To determine whether different camera trap designs improve our ability to study carrion food webs, we set up an experiment with a paired camera trap design (i.e., one horizontal and one vertical oriented) at carrion sites to monitor invertebrate and vertebrate taxa. Our objectives were to evaluate whether the camera trap designs provide different estimates of the following: (1) taxonomic richness, (2) community composition, and (3) taxonomic groups associated with each design.

## 2 Materials and Methods

### 2.1 Field Experimental Set Up

Our study was conducted in Panther Swamp National Wildlife Refuge, Yazoo, Mississippi, USA in 2017, which is characterized by an average annual precipitation of 1332.992 mm and humid subtropical climate (Wilkins 2006). We selected three sites with four plots within each site. Plots were 10 m (m) in diameter and at least 250 m apart. All the plots were composed of old‐growth, bottomland hardwood forests with similar canopy covers to avoid the strong effects of habitat on scavenger communities and decomposition rates. We deployed carrion in August, September, and November of 2017 in two plots per site. This design was part of a series of experiments focused on testing the effects of carrion from wildlife mass mortality events on ecological communities (Baruzzi, Barton, and Lashley 2023). We placed ~150 kg of wild pig ( *Sus scrofa* ) carrion per deployment randomly within each plot. Carrion was sourced from wild pig trapping efforts within the refuge, which were part of a U.S. Fish and Wildlife Service program to control for populations of this invasive species and followed the agency's internal protocols and complied with relevant federal guidelines.

**FIGURE 1:**
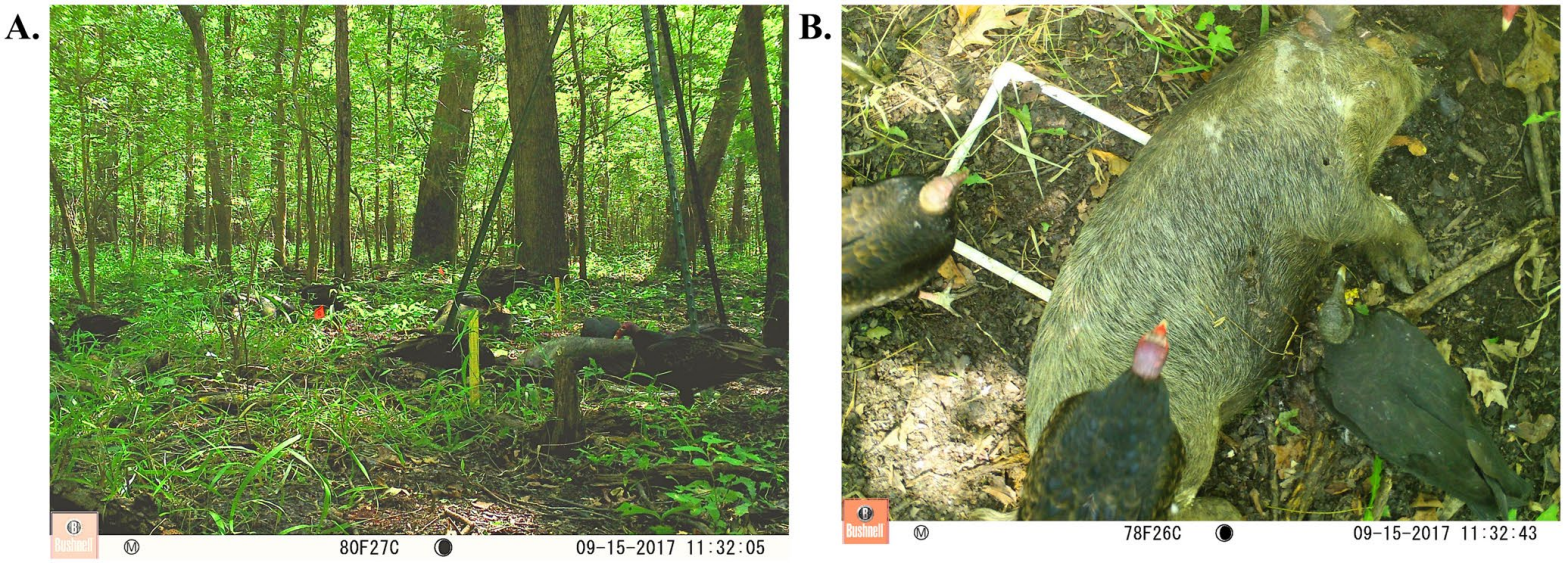
Camera trap pictures from the two designs tested. (A) Image taken with the horizontal camera design. (B) Image taken with the vertical camera design. Pictures were taken at the same site, date, and time.

Cameras (Bushnell Trophy Cam, Bushnell Outdoor Products, Overland Park, KS) and carrion were deployed on the same day. We set up one horizontal and vertical oriented camera per plot. While multiple sites likely fell into the same home range of some scavengers (e.g., turkey and black vultures; Holland et al. 2017), pairing cameras within the same plot makes any resulting spatial non‐independence expected to be similar across treatments and therefore unlikely to systematically bias our results. Horizontal cameras were placed approximately 0.5 m above ground on a tree and around 2 m from the plot's perimeter to capture the full plot area (i.e., between 5 and 8 m from each carcass). This distance was essential to ensure that the carrion was fully within the camera's field of view. Placing the cameras closer, given their mounting height and upward viewing angle, would have resulted in the carcass being partially or entirely outside the frame. Within each plot, vertical cameras were placed on a tripod made of two crossing t‐posts approximately 3 m above one individual carrion that was secured to the ground with steel garden staples. As the camera angle was associated with the distance to carrion, our designs reflected a combined consideration of orientation and proximity (Figure 1). We used the same settings in both designs: cameras were triggered by motion sensors with a 1‐min delay between triggers, and were additionally set to picture mode to take time‐lapse images once per hour. Motion sensor sensitivity was set to “Normal.” Due to the large number of camera triggers associated with baiting and the high activity of wildlife, cameras were set up to take a single image per trigger. Cameras were checked every 2 weeks. The footage review consisted of recording each detection identified to the lowest possible taxonomic unit, along with the number of individuals present, location, date, and time. Each image was identified by one reviewer and independently confirmed by another. One horizontal camera malfunctioned during the first month of data collection, so we excluded data from both the affected camera and its paired vertical counterpart to maintain a balanced design.

### 2.2 Analysis

To address our objectives, we selected any detections within the first 2 weeks after carrion deployment, which is the approximate average decomposition time of wild pigs' carrion (see Chin et al. 2008; Schlosberg et al. 2025). To determine whether the two camera trap designs produced different taxonomic richness estimates, we conducted a generalized linear mixed‐effect model with a Conway‐Maxwell Poisson distribution using the glmmTMB package (Brooks et al. 2017). Diagnostic tests using the DHARMa package (Hartig 2024) revealed underdispersion using the Poisson model, therefore the Conway‐Maxwell Poisson distribution was used as a flexible alternative suitable for count data with varied dispersion levels (Sellers and Shmueli 2010). We used camera trap design and month as fixed effects, and plot nested within site as a random intercept to account for spatial correlation. Since our study was conducted for only 3 months, we treated month as a fixed effect rather than a random effect due to the limited number of levels. Model fit was evaluated using simulated residual diagnostics implemented in the DHARMa package (Hartig 2024). In this analysis, taxonomic richness generally refers to the lowest identifiable taxonomic unit. We made an exception for adult blow flies and blow fly larvae, which were grouped separately to reflect their distinct functional roles within the carrion food web, as the larvae are responsible for consuming carrion tissue (Shah et al. 2015).

Community differences between the two camera trap designs were visualized using a non‐metric multidimensional scaling based on Jaccard distance, with presence or absence as the binary response, through the vegan package (Oksanen et al. 2024). Since it was not feasible to accurately estimate fly larvae abundances, particularly with the horizontal camera trap design, as larvae are small and often occur aggregated within carrion masses, we used only presence‐absence data and did not conduct a further abundance analysis. For the same reason, we did not define independent observations using a specific time interval, as this would not affect the presence–absence matrix used in our analyses. We tested for significant differences in the carrion community composition between camera trap designs by running a permutational multivariate analysis of variance (perMANOVA) using the adonis2 function in the vegan package (Oksanen et al. 2024), with strata = interaction (Site, Month) to account for non‐independence across site‐month combinations. We confirmed the assumption of homogeneity of dispersion was met using the betadisper function in the vegan package (Oksanen et al. 2024) and running a permutation test with 999 permutations, resulting in a nonsignificant difference in dispersion between camera designs (*F* = 0.86, *p* = 0.36).

Lastly, to compare which taxa were significantly associated with the horizontal and vertical camera design, we ran an indicator species analysis with 999 permutations on taxa presence‐absence using the indicspecies package (Cáceres and Legendre 2009). In this and all the previous analyses, we did not group images within a set time interval to define independent events (e.g., greatest group size within 30 min) because they were based on presence–absence and taxonomic richness. As a result, the structure of the presence–absence matrix and taxonomic richness dataset would remain the same regardless of abundance per image. We used a significance level of 0.05 for all analyses. Corresponding figures were created using ggplot2 (Wickham 2016) and ggpubr (Kassambara 2023). All analyses and associated figures were conducted in program R version 4.5.2 (R Core Team 2025, Vienna, Austria).

## 3 Results

The horizontal camera design captured ~5 times more total observations (133,833) than the vertical design (26,706), driven mainly by high vulture detections. We detected a total of 19 taxa: 11 in the vertical design and 15 in the horizontal design. Five taxa were invertebrates (adult blow fly, carrion beetle [*Silphidae*], blow fly larvae, red spotted purple butterfly [ *Limenitis arthemis* , *n* = 2], and spider [*Araneae*, *n* = 2]). Fourteen taxa were vertebrates, with 11 identifiable to the species level (black vulture [ *Coragyps atratus* , *n* = 135,642], turkey vulture [ *Cathartes aura* , *n* = 24,018], nine‐banded armadillo [ *Dasypus novemcinctus* , *n* = 1], coyote [ *Canis latrans* , *n* = 11], white‐tailed deer [ *Odocoileus virginianus* , *n* = 19], feral domestic dog [ *Canis familiaris* , *n* = 3], eastern fox squirrel [ *Sciurus niger* , *n* = 5], Virginia opossum [ *Didelphis virginiana* , *n* = 558], wild pig [ *Sus scrofa* , *n* = 205], raccoon [ *Procyon lotor* , *n* = 22], eastern woodrat [ *Neotoma floridana* , *n* = 1]). Three of these vertebrates were not distinguishable to species, but the number of detections for unidentifiable species was low (i.e., bird [*Aves*; *n* = 1], mouse [*Peromyscus* spp.; *n* = 2], squirrel [*Sciurus* spp.; *n* = 3]). For all taxa listed, the *n* represents the total number of observations. We are not reporting number of observations for blow flies and carrion beetles because we did not count individuals in these groups (see analysis methods for details).

**FIGURE 2:**
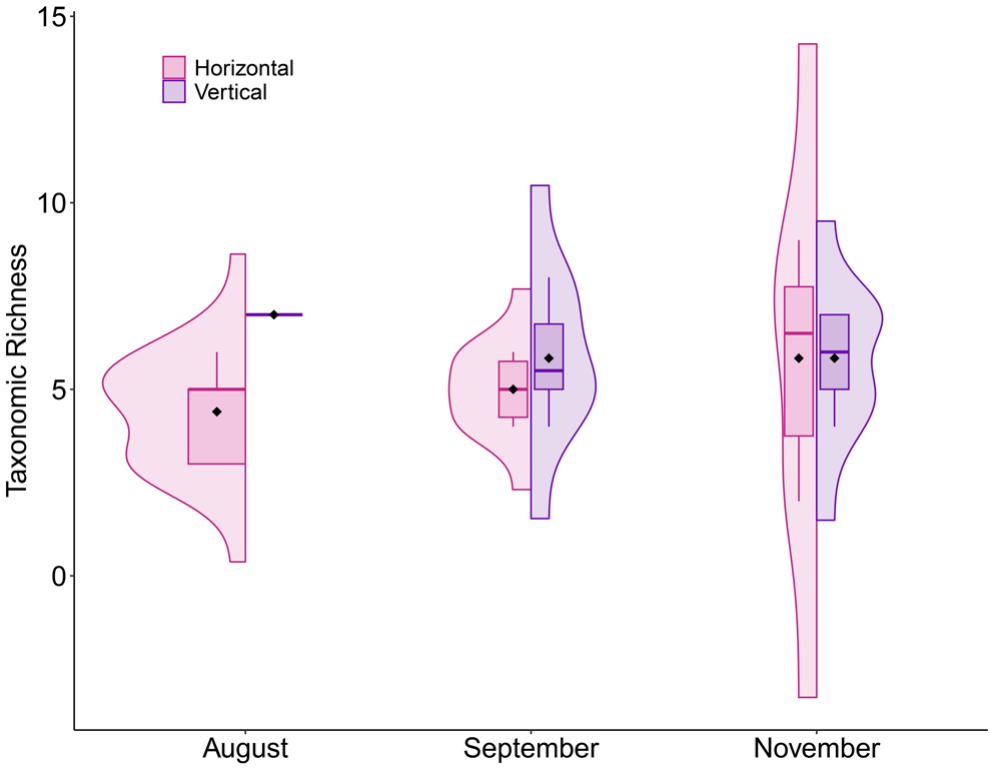
Split violin visualizing the mean taxa richness for each combination of month and camera design. The taxa richness for all vertical cameras in August resulted in seven taxa for all observations, hence why the boxplot has no interquartile range and the box is comprised of a single line.

**FIGURE 3:**
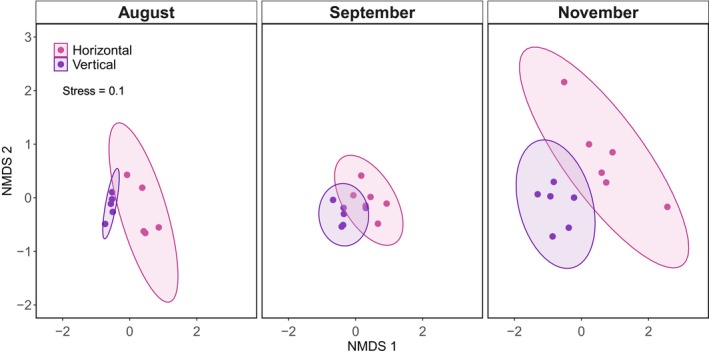
Non‐metric multidimensional scaling ordination of the horizontal and vertical camera designs by month, using Jaccard distances. The ellipses represent the 95% confidence regions around the group centroids. The stress value for two dimensions (*k* = 2) = 0.10; therefore, it is a fair representation of the original distance matrix.

Taxa richness was significantly higher in the vertical camera design (*Z* = 2.12, β = 0.19 ± 0.09, *p* = 0.034; Figure 2 and Table 1). Additionally, the community composition detected by the two camera trap designs was significantly different (*F* = 12.13, df = (1, 32), *R* ^*2*^ = 0.27, *p* = 0.001; Figure 3). The indicator species analysis further highlighted this difference by showing that white‐tailed deer (*IndVal* = 0.64, *p* = 0.007) were significantly associated with the horizontal camera trap design. In contrast, adult blow fly (*IndVal* = 0.94, *p* = 0.001), blow fly larvae (*IndVal* = 0.91, *p* = 0.001), and carrion beetle (*IndVal* = 0.73, *p* = 0.002), three of the five invertebrates detected during this study, were significantly associated with the vertical camera trap design. Obligate vertebrate scavengers (i.e., black vulture and turkey vulture) were not associated with either camera trap design.

**TABLE 1:** The mean, standard deviation, standard error, minimum, and maximum taxa richness by month and camera design.

Taxa richness						
Month	Design	Mean	Standard deviation	Standard error	Minimum	Maximum
August	Horizontal	4.40	1.34	0.60	3.00	6.00
August	Vertical	7.00	0.00	0.00	7.00	7.00
September	Horizontal	5.00	0.89	0.37	4.00	6.00
September	Vertical	5.83	1.47	0.60	4.00	8.00
November	Horizontal	5.83	2.79	1.14	2.00	9.00
November	Vertical	5.83	1.33	0.54	4.00	7.00

## 4 Discussion

We observed a significant effect of camera trap design on the detected carrion food web composition. The vertical camera trap design effectively captured invertebrates, which supports findings from previous studies on this alternative sampling method (Collett and Fisher 2017; Gao et al. 2024). The horizontal camera trap design captured more white‐tailed deer, likely due to the wider detection angle, suggesting that the use of horizontal cameras may increase detections of non‐scavenger taxa thanks to their effectiveness at detecting medium‐ to large‐sized vertebrates (Meek et al. 2016; Fancourt et al. 2017; Moore, Champney, et al. 2020). There was no association with either design for both black and turkey vultures despite these two species having the highest number of observations. This pattern is likely driven by high detection probability; as large, abundant obligate scavengers, they were consistently detected regardless of camera design. As such, a paired horizontal and vertical camera trap design at carrion sites may increase detections of the full carrion food web across sizes, emphasizing that researchers studying complex, multi‐taxa systems like carrion food webs should carefully consider whether a single design or a paired approach best aligns with their research objectives.

Previous research on carrion food webs has used horizontal camera trap design coupled with other arthropod sampling methods, such as pitfall and sticky traps, sweep nets, and manual collection (Gu et al. 2014; von Hoermann et al. 2021). These methods allow for easier species identification, as arthropods often need to be captured for identification at the species level; for example, in our study, the only arthropod identified to the species level was the distinctly marked red‐spotted purple butterfly ( *Limenitis arthemis* ). However, these other more traditional methods also have drawbacks. In our study, we additionally placed sticky traps at our carrion sites to complement our data collection, but most were missing within a week after carrion deployment, likely due to contact with vertebrate scavengers. Moreover, invertebrate species vary in their attraction to sticky traps, as factors like color and placement of the traps can influence which species are captured (Ikemoto et al. 2021; Rubio‐Aragón et al. 2024). Further, a study comparing pitfall traps to vertical camera traps found that cameras detected twice the number of arthropod taxa each day (Collett and Fisher 2017), highlighting an advantage of camera traps. In addition, camera traps can provide information on arthropod activity (e.g., hours active and activity peak; Johnson et al. 2020) that these other trapping methods do not. Thus, our vertical camera design can be a valid alternative or complement to traditional arthropod sampling methods, depending on specific research objectives.

Although our study was specifically designed to monitor the carrion food web, not all the taxa detected were directly associated with carrion. White‐tailed deer, for example, were significantly associated with the horizontal camera trap design, but are generally considered secondary consumers in the carrion food web (but see Meckel et al. 2018). Herbivores can be attracted to carrion sites as carcasses enrich the soil with nutrients (Towne 2000; Danell et al. 2002; Macdonald et al. 2014), thereby increasing plant palatability to herbivores (Yang 2008; Baruzzi, Barton, Cove, et al. 2023). However, observing these top‐down effects of carrion on herbivores requires a more extended monitoring period, as carcasses must first decompose and release nutrients into the soil before being absorbed by the plants. Given our monitoring took place within the first 2 weeks of carcass decomposition, we believe deer detections were opportunistic and resulted from the wide detection angle of the horizontal camera. Notably, wild pigs were equally detected in both camera trap designs, but the vertical design additionally captured a few individuals wallowing nearby decomposing carcasses, suggesting that the vertical design could offer insights into fine‐scale behaviors that may be undetectable at greater distances. Although some behaviors, such as vulture sunbathing and roosting, are more likely to be detected by a horizontal design. Conversely, the horizontal design's wider field of view made it more suitable for capturing broader behaviors, such as vulture sunbathing and roosting, which occur away from the immediate carcass.

Given that species detections can be affected by camera trap designs, we suggest that research objectives guide the choice of camera design. Since photographic identification can be time consuming, using a single camera design may be preferred when a paired set up is not necessary. For example, if the goal is to detect invertebrates and their activity, researchers may consider using only a vertical design. Similarly, if the goal is to monitor medium‐ to large‐bodied vertebrates across the carrion food‐web, a horizontal design may be more suitable. This may hold true for systems beyond carrion food webs and is critical to explore in systems where vertebrates and invertebrates play interacting roles in ecosystem functioning.

## Author Contributions

**Annesha Lahiri:** writing – original draft (equal), writing – review and editing (equal). **Kathleen A. Carey:** data curation (equal), formal analysis (lead), visualization (lead), writing – original draft (equal), writing – review and editing (equal). **Marcus A. Lashley:** conceptualization (equal), funding acquisition (equal), investigation (equal), methodology (equal), supervision (lead), writing – review and editing (equal). **Michael V. Cove:** conceptualization (equal), methodology (equal), writing – review and editing (equal). **Brandon T. Barton:** conceptualization (equal), funding acquisition (equal), methodology (equal), resources (equal), writing – review and editing (equal). **Carolina Baruzzi:** conceptualization (equal), data curation (equal), formal analysis (equal), investigation (lead), methodology (equal), project administration (equal), writing – original draft (equal), writing – review and editing (equal).

## Funding

This research was funded by startup funds from Dr. Marcus Lashley from the Forestry and Wildlife Research Center, the Department of Wildlife, Fisheries, and Aquaculture and from Dr. Brandon Barton from the Department of Biological Sciences at Mississippi State University.

## Conflicts of Interest

The authors declare no conflicts of interest.

## Data Availability

The dataset and R code supporting the findings of this study are publicly available through a GitHub repository at: https://github.com/Kathleencarey/Carrion_Camera.
